# Evaluation of Model and Process Optimization for the Treatment of Drilling Wastewater Using Electrocoagulation

**DOI:** 10.3390/molecules30051064

**Published:** 2025-02-26

**Authors:** Muhammed Kamil Öden

**Affiliations:** Department of Environmental Protection Technology, Sarayönü Vocational High School, Selcuk University, 42430 Konya, Türkiye; muhammedkoden@selcuk.edu.tr; Tel.: +90-332-617-2800

**Keywords:** wastewater treatment, response surface method, drilling wastewater, Fe–Cu electrode, electrocoagulation

## Abstract

The extraction of underground resources has accelerated globally, in response to the demands of advancing technology and the rapidly growing population. The increase in drilling activities has caused an increase in environmental pollution problems caused by waste generated during drilling activities, namely drilling sludge and drilling wastewater. In this study, the treatability of wastewater generated during drilling operations in a basin, where an underground gas storage area was created, was investigated using an electrocoagulation (EC) process, using different electrode pairs. The removal efficiencies of the pollution parameters were determined using the response surface method. The wastewater parameters included different organic and inorganic pollutants, such as sodium, chloride, magnesium, and chemical oxygen demand (COD). The concentrations of sodium, chloride, and COD in drilling industry wastewater were found to be very high, at 128,567, 185,292, and 7500 mg/L, respectively. The data obtained in this study yielded a removal efficiency of approximately 65% and above. Sodium achieved the highest pollutant removal efficiency of 85% and above. The statistical values were interpreted for all the pollutants and the suitability of second-degree regression was observed.

## 1. Introduction

Access to clean and fresh water is a fundamental necessity for human civilization. Climate change and freshwater scarcity have become some of the most significant global challenges faced by humanity in the 21st century [1]. Water is also one of the renewable resources necessary for maintaining all life forms, food production, economic development, industry, agriculture, and general well-being. A country’s surface water and underground water resources are either used or polluted during industrial activities, such as agriculture, hydroelectric production, animal husbandry, forestry, fishing, and maritime activities [2]. Up to 5 million gallons of water are used during the drilling and fracturing of a single well and, afterwards, water flows back to the surface and becomes drilling and fracturing waste. Moreover, the wastewater produced flows out of the well, together with oil or gas, throughout most of the well’s productive lifetime [3,4].

Drilling fluid is a chemically prepared mixture, with specific properties, for circulation during the rotary drilling process [5,6]. The global production of drilling waste (DW) exceeds 25 × 10^6^ m^3^ [7] and wastewater discharge from oil and gas (O&G) operations account for 14.5 × 10^9^ m^3^ [8] annually. Water is also used during and after drilling operations to access oil, gas, and water. The drilling and hydraulic fracturing of O&G wells result in several environmental impacts, which have become a major concern worldwide. Key environmental impacts include groundwater quality degradation, the management of the wastewater produced, and the generation of drilling sludge during drilling and fracturing processes [9]. Waste drilling fluid and mud contain a high solids content, minerals, drill cuttings, and organic materials, etc. Therefore, the direct discharge of drilling wastewater (wastewater and waste drilling fluid) is strictly prohibited, due to the high levels of organic matter, salinity, and solids that are harmful to the environment and public health [10]. Hence, implementing more environmentally friendly and efficient wastewater treatment methods is necessary. Organic and inorganic pollutants, such as dyes, heavy metal ions, salts, and surfactants, present in wastewater have adverse effects on aquatic ecosystems and human health [11,12,13]. Various methods are used to treat drilling wastewater, including processes such as mechanical vapor recompression (MVR) [14], biodegradation [15], electrocoagulation [16], activated sludge [17], the Fenton process [18], adsorption [19], and coagulation [20]. Among the traditional treatment techniques, the electrochemical technique has achieved significant gains in wastewater treatment, due to its unequaled advantages, such as its easy design and operation, cost effectiveness, and high pollutant removal efficiency [21,22].

Electrocoagulation (EC) is an electrochemical-based technique that produces coagulant species in a reactor from an electrode solution made of anodes, usually made of iron, copper, or aluminum, which degrade over time. It destabilizes suspended, dissolved, or emulsified pollutants by applying an electric current to wastewater or water. It has the potential to remove a wide range of pollutants, including organic and inorganic pollutants, from different types of wastewater [23].

Studies on the application of electrocoagulation as an environmentally friendly technology for the removal of certain pollutants in waste water have increased in recent years [24]. However, not enough studies have focused on pollutants in drilling wastewater. Therefore, a systematic study on the removal of pollutants from drilling wastewater using electrocoagulation is necessary and innovative, considering both environmental protection and industrialization requirements.

Additionally, the electrocoagulation process can remove the smallest colloidal particles, which is not possible using the traditional flocculation/coagulation method [25,26]. Electrocoagulation (EC) is one of the best wastewater treatment processes. The principle of EC is based on the electrical dissolution of metal electrodes and their use as coagulants in the formation of ions. Metal ions are produced at the anode and hydrogen gas is released at the cathode. The hydrogen gas bubbles formed at the cathode cause flocculated particles to float to the water’s surface [27,28]. The advantages of EC, including its flexibility, ease of use, no need for the addition of any chemicals, short ramp-up time, ease of monitoring, and multipollutant capability, have spurred research interest. Various environmentally friendly processes and indexes have been used in many environmental research studies. Contributing to environmental protection is among the primary goals of this research study [29,30,31].

The present study investigated the treatment efficiency of the EC process in regard to drilling wastewater. Process efficiency was evaluated through analyzing its effect on various operating parameters (current density, electrolysis time, and initial pH), in order to find the most adequate and favorable operating conditions for the use of an iron and copper electrode couple. The operating parameters were the removal efficiencies in terms of sodium, chloride, magnesium, and chemical oxygen demand (COD), achieved using the EC process. In Turkey, for the first time, the wastewater released during drilling around the Salt Lake for natural gas storage was used to develop a mathematical model with the response surface methodology (RSM) approach, using the central composite design (CCD), and to examine the interactive effects of the investigated parameters.

## 2. Results and Discussion

### 2.1. Statistical Analysis and Experimental Models

In this study, the response surface methodology (RSM) was used to optimize the EC process, using three experimental variables and factors. The RSM uses mathematical and statistical techniques to help optimize processes. The RSM uses an experimental model, such as the central composite design (CCD), to apply the least squares method to a model [32,33]. The RSM determined the process responses, such as the chloride, magnesium, and sodium removal efficiencies, using three variables (the initial pH, current density, and contact time). Table 1 shows the levels of these three variables determined in this study. A second-order polynomial response surface model was used to fit the experimental data, using data from the central composite design process (Equation (1)). The electrocoagulation process results were estimated using this model relationship.

**Table 1:** Independent variables and code levels for the EC process.

Coded Factors	Symbol and Variables		
	K_1_: Current Density (mA/cm^2^)	K_2_: Electrolysis Time (min)	K_3_: Initial pH
−1	16	10	3
0	32	20	6
1	48	30	10

(1) $$Y=\beta_{0}+\beta_{1}K_{1}+\beta_{2}K_{2}+\beta_{3}K_{3}+\beta_{11}{K_{1}}^{2}+\beta_{22}{K_{2}}^{2}+\beta_{33}{K_{3}}^{2}+\beta_{12}K_{1}K_{2}+\beta_{13}K_{1}K_{3}+\beta_{23}K_{2}K_{3}$$

K_1_, K_2_, and K_3_ represent the encoded levels of the design variables (K_1_: density; K_2_: contact time, and K_3_: pH). The interactions are denoted as beta12, beta13, and beta23; the quadratic coefficients as beta11, beta22, and beta33; the linear coefficients as beta1, beta2, and beta3; the intercept with beta0; and the set of regression coefficients as β (beta). Y stands for the predicted response (% removal efficiency) (Y_1_: chloride removal efficiency; Y_2_: magnesium removal; Y_3_: sodium removal; Y_4_: COD removal; and Y_5_: sulfate removal). Only the equation for the COD and sulfate values was given. Since the model results were not meaningful, the other data were not added. The wastewater treatment results from the electrocoagulation process were defined using significant equations in the software program environment. Table 2 shows the regression equations used for the model test for the copper–iron electrode pair. The effects of the electrocoagulation process on the chloride, magnesium, and sodium removal efficiencies, in the 15 experiments, are shown in Table 3. The effects on the removal efficiencies of the studied parameters on the variables of the copper–iron electrode couple are shown in Table 3. Synergistic and antagonistic effects are indicated by positive and negative numbers before the values in the given equations [34]. The results of the analysis of variance (ANOVA) for the second-order polynomial model in terms of the response surface model are presented in Table 4. The F-test confirmed the significance of the regression results. Correlation coefficients (R^2^ and adjusted R^2^) were used to check the fit of the quadratic equations. The *p*-values obtained provide us with information on the statistical interpretation of the F-values. The suitability and model inadequacy values were as follows (see Table 4): the model’s *p*-value < 0.05 and (f-value), *p*-value > 0.05, respectively. The model was statistically significant if the Prob > F value was between 0.003 and 0.05, or less than 0.05.

In regard to the analysis of the results for the Cu–Fe electrode couple, the R^2^ and adjusted R^2^ values were higher than 83.69% and 81.01, and 76.82% and 55.33, respectively. The values of the correlations are listed from high to low: sodium > magnesium > chloride for the electrode pair. The sum of the square values of the model were 647.575, 1739.64, and 449.082 for the Cu–Fe electrode couple during the EC process. Additionally, this research revealed that the wastewater generated during drilling contained significant amounts of chloride, sodium, and magnesium. The electrocoagulation process achieved a removal efficiency of over 80% in regard to the pollutant parameters. The electrocoagulation process, conducted using a copper–iron electrode pair, alongside the response surface methodology, demonstrated strong alignment with the statistical data. The F-value of the model ranged from between 0.02–10.56, 0.01–9.35, and 0.77–9.21. These F-values indicate that the models for the chloride, magnesium, and sodium removal efficiencies were statistically significant in regard to the electrocoagulation process. The mean square values for the Cu–Fe electrode pair were calculated as 259.692 for chloride, 45.63 for magnesium, and 64.38 for sodium.

### 2.2. Effect of Operating Variables on Chloride, Magnesium, and Sodium Removal

An electrocoagulation process was established in the laboratory to investigate chloride, magnesium, and sodium removal from drilling industry wastewater. The effects of the current density, pH, and contact time variables on the treatment were investigated during the prepared treatment process. Table 4 contains the ANOVA test results of the response surface quadratic model for the chloride, magnesium, and sodium removal at the electrode. Table 4 shows that the linear coefficients of the current density (K_1_) and pH (K_3_) had significant effects on the pollutant removal performance, as did the electrolysis time (K_2_) and pH (K_3_), on the chloride, magnesium, and sodium removal efficiency in regard to the electrode couple. The chloride, magnesium, and sodium removal were significantly affected by the second-order current density (K_1_K_1_). The copper removal in regard to the Cu–Fe electrode pair was also significantly affected by the second-order relationships of the current density and contact time (K_1_K_2_).

Regarding sodium removal, the pH (K_3_) and the coefficients of the factor K_3_K_3_ influenced the EC process (*p* > 0.05), whereas the current density (K_1_) with the electrolysis time (K_2_) and the quadratic terms of the current density and electrolysis time (K_1_K_1_) had highly significant effects on sodium removal (*p* < 0.003). In regard to chloride removal, the square terms of the current density (K_1_K_1_), reaction time (K_2_K_2_), and initial pH (K_3_K_3_) significantly affected chloride removal. Table 4 shows the response surface ANOVA results for chloride, magnesium, and sodium removal using the Cu–Fe electrode combination. Table 4 shows that the linear coefficients of the current density (K_1_) and electrolysis time (K_2_) had an effect on the chloride removal efficiency, and the linear coefficients of the current density (K_1_) and electrolysis time (K_2_) had an effect on the magnesium removal efficiency. Moreover, the electrolysis time (K_2_), current density (K_1_), and pH (K_3_) had significant effects on the sodium removal efficiency. The magnesium parameter had significant effects on the current density (K_1_), contact time (K_2_), and pH (K_3_). The second-order terms of the current density and pH had significant effects on magnesium removal in regard to the treatment with the electrode pair.

**Figure 1:**
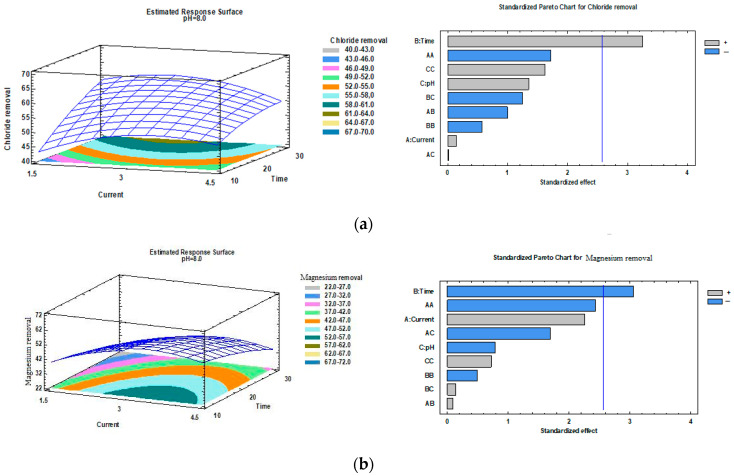

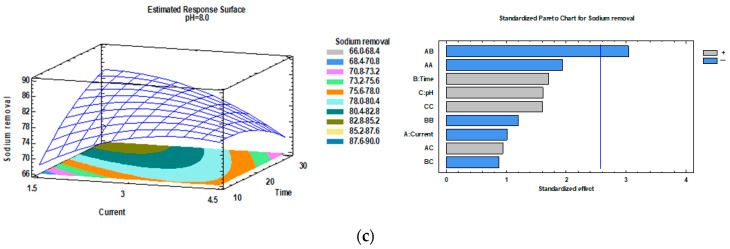
Three-dimensional graphs for (**a**) chloride, (**b**) magnesium, and (**c**) sodium in electrode couples; current density and electrolysis time versus pH.

The three-dimensional response surface graphs obtained from the experimental study are shown in Figure 1, for the Cu–Fe electrode couple. Using experimental data, three-dimensional colored graphs of the current density versus time, current density versus pH, and pH versus time, were prepared. In general, during the experimental study, it was observed that the changes in the stream density, contact time, and pH variables directly affected the pollutant removal efficiency from the wastewater at the central level. We cannot claim that no variables affected the treatment efficiency. At least two optimization variables were important for the removal of three different pollutants. The response and contour plots from the quadratic model for chloride, magnesium, and sodium removal in regard to the Cu–Fe electrode pair are shown in Figure 1. The increase in time did not significantly affect the magnesium removal efficiency. The current density had a significant effect on the removal rate. However, the time and current density increased the chloride and sodium removal efficiency. This horizontal bar graph shows the combination of factor levels that maximized the chloride, magnesium, and sodium removal across the indicated region.

The ANOVA results for the optimum data in the experiment carried out with the copper–iron electrode pair are shown in Table 5. A current density of 32 mA/cm^2^, an electrolysis time of 30 min, and a pH of 6 were the most appropriate values for chloride removal with the Cu–Fe electrode pair. According to these conditions, the estimated COD removal efficiency of the model was 81%. The chemical oxygen demand (COD) measures the amount of oxygen used in the chemical oxidation of inorganic and organic matter present in wastewater. Although the COD is not a specific compound, it is considered to be a conventional pollutant under the federal Clean Water Act and is widely used by regulators worldwide to measure the overall efficiency of wastewater treatment plants. It is also an indicator of the level of pollution in effluent and the potential environmental impact of wastewater discharge into water bodies. The COD is measured using a strong chemical oxidant in standard conditions [35]. The experimental chloride removal efficiency was found to be approximately 70% via the treatment process carried out in the optimum conditions established in regard to the experiment. These findings show that the model estimate for chloride removal is within the confidence interval. After the treatment of the wastewater generated during drilling operations, the chloride value was calculated as 55,587.6 mg/L in optimum conditions. This is quite high in relation to local discharge limits. The findings indicate that the wastewater generated by the drilling process does not meet the direct discharge limits set by the Turkish Ministry of Environment and Urbanization. According to the regulation, the highest chloride discharge limit is 15,000 mg/L, even for the chemical industry (soda production). This value is even higher than this limit. However, high chloride removal efficiencies were achieved, and the effluent became less harmful in this experimental study.

The optimum conditions for magnesium removal using an electrode pair were found to be a current density of 48 mA/cm^2^, a contact time of 10 min, and a pH of 6. The experimental removal efficiency and the removal prediction efficiency of the model for the magnesium treatment were found to be 65% and 83.28%, respectively. The optimum conditions for sodium removal in regard to the treatment carried out with the Cu-Fe electrode pair were 32 mA/cm^2^ for the current density, 30 min for the contact time, and a pH of 10. The experimental removal efficiency and the removal prediction efficiency of the model for the sodium treatment were 86.74% and 95%, respectively. The experimental values for magnesium and sodium removal were consistent with the predicted values. The sodium removal efficiency was significantly higher than that of magnesium and chloride in regard to the Cu–Fe electrode couple. Unfortunately, the sodium concentration was well above the discharge limits. Turkey’s Water Pollution Control Regulation does not include any values in terms of the discharge limits for wastewater generated during drilling. However, there are discharge limits for different parameters for many industries and activities located nearby. The treatment results obtained in this study were compared with the appropriate discharge limits.

This study obtained removal values of 70%, 65%, and 86,74% for chloride, magnesium, and sodium in regard to the copper and iron electrode couple, respectively. The parameter (chloride, magnesium, and sodium) concentrations after treatment were calculated as 55.5876; 13.195; and 17.0479 mg/L. The discharge limits for chloride and sodium parameters are provided in the regulation. However, there is no comparable value for magnesium. The removal efficiencies for the COD, sulfate, and Ca/Mg were also obtained. However, these parameters’ data were not included in this study, because they were not appropriate for the model. Removal efficiencies of 82.2%, 67.4%, and 87.5% were obtained in the optimum conditions. The experimental results from this research in regard to the removal of all the contaminants from the wastewater were consistent with the predictions and were significant. In the experimental study on pollutant removal carried out in the determined optimum conditions, a removal efficiency of over 65% was achieved for all the pollutants. A 65% removal efficiency using a single process can be considered to be very successful.

The optimum application results, detailed in Table 5, show that the pH range of the three parameters had an alkaline range. Additionally, higher efficiency values were obtained when the current density was above 32 mA/cm^2^. When the contact times were examined, the highest removal efficiencies were obtained at times between at least 10 and 30 min. The short magnesium contact time was balanced by the application of the highest current density.

Oxygen must be present and the pH value must be neutral or alkaline to achieve a reasonable reaction rate during EC treatment [36]. With a low pH, the chemical dissolution of iron can be significant, and the total iron concentration can be higher than is theoretically expected. According to the results from another study, no significant oxidation occurred at pH 5, the oxidation rate was moderate at pH 6, and very rapid oxidation occurred at pH 7–9 [37]. The applied voltage directly affects the coagulant dosage rate and the current density, which determines the bubble production rate and the size and growth of flocs, as well as affecting the removal efficiency of the electrochemical process [38,39]. In this research, pH values of 6, 6, and 10 were determined for chloride, magnesium, and sodium, respectively.

When looking at the results in Table 5 and Table 6 or searching the literature, there are many studies on the use of different electrodes in the electrocoagulation process and the use of different types of raw/synthetic wastewater. However, we seldom encounter work using raw wastewater with such high inlet pollutant concentrations as in this research study. Our research study aimed to create natural gas storage areas by taking advantage of the geological structure in the Salt Lake. The treatment of wastewater resulting from drilling operations, during the construction of these storage areas, via electrocoagulation, was investigated. This appears to be an original and new environmental study. The optimization criteria presented in Table 6 were determined as a 10–30 min contact time for chloride, magnesium, and sodium, a pH between 6 and 10, and a current value within an acceptable range.

Table 6 shows the relationship between this study and other studies in the literature. In most studies, the pH values were determined to be closer to alkaline levels, generally ranging between pH 6 and 10. In our study, pH values consistent with the literature were obtained. Real raw wastewater was used to ensure the accuracy of the data in this research. Additionally, while many studies required operation times exceeding 20 min, in our research, an operation time of 10–40 min was sufficient.

## 3. Materials and Methods

### 3.1. Characterization of Drilling Wastewater

The wastewater from drilling operations used in this study was obtained from drilling operation activities in the province of Aksaray. Wastewater samples were stored at +4 °C for up to 10 days after collection. Table 7 presents the characterization data of the raw wastewater.

Heavy metal analyzes were performed using a Perkin Elmer (Shelton, CT, USA) ICP Mass Spectrometry (ICP-MS) NexION 350×. The control operations were carried out with a Hach Lange Dr3900. Other analyses were performed according to the standard methods [48]. All chemicals used were of analytical purity. Equation (2) was used to compute the percentage pollutant removal efficiency (Y) as follows [49];

(2) $$\;Y,\%=\frac{C_{i}-C_{f}}{C_{i}}\times 100\%$$

(C_i_ and C_f_ are the concentrations of the pollutant at the beginning and end in mg/L, respectively).

### 3.2. Experimental Details and Procedure

**Figure 2:**
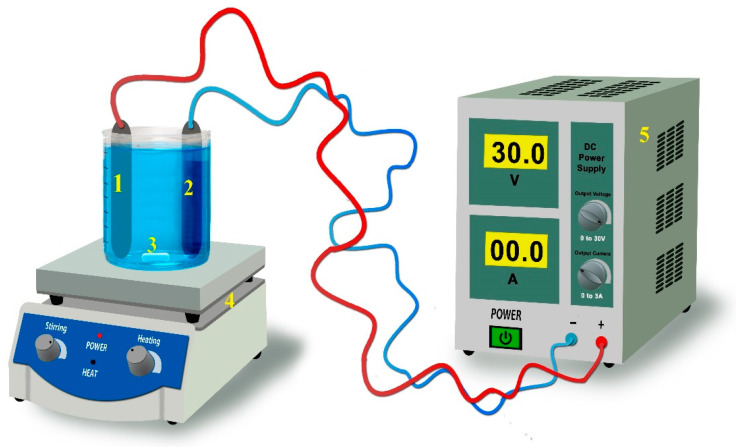
Diagram of EC in the laboratory (1: reactor unit; 2: electrode; 3: stir bar; 4: magnetic stirring equipment; 5: DC power supply).

Wastewater is generated during drilling, boring, and piping operations. The wastewater from drilling operations used in this study was obtained from drilling operation activities in the province of Aksaray. Since the activity area is very close to the Salt Lake, certain parameters, such as chloride and sodium levels, are very high. Two electrodes (one anode and one cathode) were placed vertically, 3–4 cm apart, into the reactor, containing wastewater. A schematic diagram of the electrocoagulation process is shown in Figure 2. An EC reactor, at a laboratory scale, was made from a piece of Plexiglas, with a length of 13 cm and a diameter of 9 cm. In regard to the laboratory experiment, iron and copper electrodes were prepared and used, consisting of an anode and a cathode, which were 11.5 cm high, 6 cm wide, and 0.1 cm thick. The total impact area of the electrodes was calculated as 80–90 cm^2^. The electrode materials used in this research were obtained commercially, without a brand or model. The electrode materials consisted of 100% pure raw metal. The obtained electrode pairs were studied in control conditions, so that their properties would not affect the results of this study. A Mervesan 305D II power supply was used to supply a direct current to the reactor. Moreover, 500 mL of effluent was used in this study, as well as 500 mL of wastewater. During the experiments, the wastewater was mixed in the reactor, using a magnetic stirring device, operating at 200 rpm.

The surfaces of the electrodes were cleaned with acetone before the electrocoagulation tests. The electrodes were soaked for at least 5 min in a cleaning solution (200 mL of 2.8% C_6_H_12_N_4_ and 100 mL of 35% HCl). This was followed by rinsing them with distilled water and drying them with a disinfecting solution at 105 °C. The response surface method (RSM) was used to optimize the current density (16–48 mA/cm^2^), pH (5–9), and reaction time (10–30 min), which influenced the EC process. There was no need to add NaCl during the experiments, due to the high conductivity of the solution. The purified samples were removed using a pipette and analyzed, according to standard methods, after a two-hour precipitation period, following each experimental set. The COD, sulfate, chloride, sodium, magnesium, and Ca/Mg analyses were performed using samples taken from the clear phase.

### 3.3. Electrocoagulation Process

The basic principles of the electrocoagulation process are derived from the electrolysis process. The basic principles of electrolysis were first defined and developed by Michael Faraday [50]. Typically, there are three main steps: electro-oxidation, electroflotation, and electrocoagulation. The operations of electro-flotation and electrocoagulation are advantageous in regard to the remediation of wastewater resources [51]. During the electrocoagulation process, a direct current is applied between the metal electrodes (such as iron, aluminum, platinum, copper, etc.) that are submerged in the contaminated wastewater. The metal cations generated within the reactor produce hydroxide that dissolves and interacts with the metal electrodes, which helps remove pollutants [52]. Several metals are suitable for use as electrodes; iron (Fe) and copper (Cu) are of particular interest, due to the low cost and high valency of the cation produced [53]. The EC process involves the in situ production of coagulating ions, during three consecutive phases: (i) electrolytic oxidation (generally a different electrode), (ii) stabilization and particulate suspension, and (iii) floc formation. The mechanism that occurs in the interior of an EC reactor that includes electrodes made of metal M can be described in terms of the chemical reactions, as follows:

At the anode:

M_(s)_ → M_(aq)_^n+^ + *n*e^−^ (3)

2H_2_O_(l)_ → 4H^+^_(aq)_ + O_2(g)_ + 4e^−^ (4)

At the cathode:

M_(aq)_^n+^ + *n*e^−^ → M_(s)_ (5)

2H_2_O_(l)_ + 2e^−^ → H_2(g)_ + 2OH^−^ (6)

These hydroxides/polyhydroxymetallic complexes show a strong affinity toward dispersed ions and particles, causing them to coagulate. Gases are released, causing the coagulated particles to rise [54,55].

The efficiency of the EC process depends on the harmonious operation of various components. Wastewater treatment can be significantly improved by fine-tuning key operational parameters. These parameters include the electrode material, current density, and pH of the wastewater. Adjusting the current density affects the rate of electrochemical reactions and, thus, the formation of coagulants, which are critical for contaminant removal. Similarly, the choice of electrode material is critical, because different materials have unique electrochemical properties that affect the efficiency of the EC process [56]. Various electrode materials, such as carbon (C), mild steel (MS), stainless steel (SS), aluminum (Al), silver (Ag), iron (Fe), nickel (Ni), and copper (Cu), have been investigated for EC applications [57]. Most EC studies have mainly used iron, stainless steel, and aluminum electrodes, with an increasing number of studies and purification successes achieved when investigating copper and nickel as electrodes [40]. It is assumed that the reduction products produced are gradually adsorbed on the copper electrode surface and are too stable to be reduced electrochemically at low reduction potentials [58].

### 3.4. Experimental Design and Statistical Analysis

The following inputs or variables must be considered when considering the potential effectiveness of an electrocoagulation–electroflotation (ECF) reactor: the wastewater type, pH, current density, the type of metal electrodes (aluminum, steel, iron, etc.), the number of electrodes, the size of the electrodes, and the metal configuration. These variables affect the overall treatment time, kinetics, and measured pollutant removal efficiency [59].

During electrocoagulation, the contact time is directly related to the current density. Increasing the contact time increases the electricity consumption. In this respect, the feasibility and applicability of this study are directly related to the optimization criteria for the process [12]. However, as the experimental study progresses, the concentration of aqueous metal oxides rapidly increases, while the contaminants in the solution decrease. As a result, the removal rate becomes constant after a certain period of time [60].

The optimization process was performed using a central composite design, in the Statgraphics Centurion XVI.I software 16.1 program environment. The RSM can be used to assess the main (K_1_, K_2_, and K_3_), interactive (K_1_K_2_, K_1_K_3_, and K_2_K_3_), and curvature effects (K_1_^2^, K_2_^2^, and K_3_^2^). Fifteen discrete EC processes were carried out with the experimental set created using the program, and the effects of the electric stream (K_1_: 0–48 mA/cm^2^), operation time (K_2_: 0–30 min), and pH values (K_3_: 0–10), on the changes in the removal efficiency of the pollutant parameters, were determined. Table 1 details these parameters and the relevant levels.

The statistical analysis of the data and the analysis of variance (ANOVA) were carried out using the Statgraphics Centurion XVI.I program 16.1. Fisher’s F-test was used to estimate the statistical significance. The model’s suitability was checked using the correlation coefficient (R^2^). This was obtained by using the corresponding probability values produced using the same software. Three-dimensional graphs were plotted depending on how the independent factors affected each other. In addition, shape plots were developed as a function of these factors.

## 4. Conclusions

This research investigated the removal efficiency of chloride, magnesium, and sodium, which are present in high concentrations in wastewater generated during drilling processes, using the electrocoagulation process, with a copper–iron electrode couple. The response surface methodology (RSM) was utilized to evaluate the removal efficiency in terms of these parameters. The primary criteria in this study included the type of electrode, electrolysis time, wastewater pH, current density, and conductivity, and their effects on pollutant removal. RSM optimization was applied to determine which parameters were statistically significant and whether there were any interactions between the parameters in regard to the electrocoagulation process.

The ANOVA results showed high correlation coefficient (R^2^) and adjusted R^2^ values, indicating that the second-order regression model fit the experimental data satisfactorily. For the quality of this study’s suitability, the model’s *p*-value was expected to be less than 0.05, and the f-value was expected to be greater than 0.05. The difference between the estimated correlation coefficient (R^2^ pred) and the adjusted correlation coefficient (R^2^adj) should not be more than 20%. The proposed model demonstrated a strong fit with the experimental data. Increasing the operation time in regard to the presence of the copper and iron electrode couple in the electrocoagulation reactor positively impacted the pollutant removal efficiency. The conclusions based on results of the experiments in this study were as follows:

- Using the Cu-Fe electrode pair under optimum process conditions, the removal efficiencies were calculated as 70.65% for chloride, 65.48% for magnesium, and 86.74% for sodium.
- In the EC process using Cu-Fe electrodes, the optimum pH was determined to be 6 for chloride and magnesium removal and 10 for sodium removal.
- The current densities during pollutant removal ranged from 32 to 48 mA/cm^2^, with an increase in the current density improving the treatment up to a certain level.

Electrocoagulation (EC) is a popular wastewater treatment alternative that has been extensively studied in regard to a wide range of wastewater types, due to its versatility, ease of installation, small environmental footprint, and environmentally friendliness. The main factors affecting the EC process are the type of electrode, the distance between the electrodes, the applied current density, the initial pH, the conductivity of the electrolyte, and the process time. Therefore, operational variables should be optimized in all EC studies [61]. The contact time found in most studies varies between 10 and 120 min and the applied current ranges from 3 to 300 A/m^2^. When the current density increases, the coagulant release rate also increases, and the contact time can be reduced. It has been observed that the operation of the process is independent of the initial pH of the wastewater, since the system’s pH is neutral or fixed throughout the process. EC combined with other treatment techniques can increase treatment efficiency and help reduce the operating time compared with EC alone. Sludge formation is the biggest problem associated with the EC process. This problem can be overcome by using the produced sludge, especially in sectors such as those that use building blocks, filler material, and concrete. Metal recovery from EC sludge via chemical washing, thermal, and other processes, can also manage sludge. A pilot-scale EC process can remove approximately 50–90% of COD and inorganic pollutants from industrial wastewater.

This study highlights the importance of both the initial use of the electrocoagulation process and the application of the RSM model to treat drilling wastewater in the Aksaray region. The use of real wastewater for the experiments further enhances the value of the findings.

## Figures and Tables

**Table 2:** Representation of the regression equation for the electrode (Cu-Fe couple).

Electrode	Parameters	Equations
Cu-Fe Couple	Chloride removal (%)	Y_1_ = 44.4408 + 15.4186* K_1_ + 2.89592* K_2_ − 12.414* K_3_ − 1.97352* K_1_^2^ − 0.165667* K_1_* K_2_ − 0.0108333* K_1_* K_3_ − 0.0148292* K_2_^2^ − 0.1545* K_2_* K_3_ + 1.0449* K_3_^2^
	Magnesium removal. %	Y_2_ = 15.2625 + 46.635* K_1_ − 0.333125* K_2_ − 6.69437* K_3_ − 4.30444* K_1_^2^ + 0.0251667* K_1_* K_2_ − 2.1575* K_1_* K_3_ − 0.019675* K_2_^2^ + 0.0275* K_2_* K_3_ + 0.721875* K_3_^2^
	Sodium removal (%)	Y_3_ = 74.55 + 12.3392* K_1_ + 3.01863* K_2_ − 11.8212* K_2_ − 1.72056* K_1_^2^ − 0.387167* K_1_* K_2_ + 0.601667* K_1_* K_3_ − 0.0238625* K_2_^2^ − 0.084* K_2_* K_3_ + 0.799063* K_3_^2^
	COD removal. %	Y_4_ = 79.425 − 1.01583* K_1_ − 0.128875* K_2_ + 1.41* K_3_ + 0.325* K_1_^2^ − 0.0141667* K_1_* K_2_ − 0.085* K_1_* K_3_ − 0.0028875* K_2_^2^ + 0.0365* K_2_* K_3_ − 0.147812* K_3_^2^
	Sulfate removal (%)	Y_5_ = 45.22 + 9.36167* K_1_ − 0.00175* K_2_ + 1.68125* K_3_ − 0.766111* K_1_^2^ − 0.0365* K_1_* K_2_ − 0.460833* K_1_* K_3_ + 0.0009125* K_2_^2^ + 0.002875* K_2_* K_3_ − 0.0221875* K_3_^2^

**Table 3:** Relationship between experimental data and model data for removal efficiency during process.

				Cu-Fe Couple					
Run	K_1_:j	K_2_:t	K_3_:pH	Y_1_: Chloride Removal (%)		Y_2_: Magnesium Removal (%)		Y_3_: Sodium Removal (%)	
	mA/cm^2^	min	-	Exp.	Pred.	Exp.	Pred.	Exp.	Pred.
1	16	10	6	40.63	43.1912	43.11	39.5637	66.25	68.25
2	48	10	6	46.94	48.6937	46.83	50.9637	74.25	77.1225
3	16	30	6	61.31	59.5562	26.45	22.3162	87.35	84.4775
4	48	30	6	57.68	55.1187	31.68	35.2262	72.12	70.12
5	16	20	3	51.84	54.64	32.23	31.4562	79.41	81.5737
6	48	20	3	51.63	55.2375	65.01	56.5562	73.93	75.2212
7	16	20	10	63.04	59.4325	31.68	40.1337	83.61	82.3187
8	48	20	10	62.7	59.9	38.57	39.3437	85.35	83.1862
9	32	10	3	54.47	49.1088	56.2	60.52	80.06	75.8963
10	32	30	3	67.73	66.6838	38.02	42.9275	83.16	83.8688
11	32	10	10	58.97	60.0162	60.06	55.1525	84.32	83.6112
12	32	30	10	59.87	65.2313	44.08	39.76	80.7	84.8638
13	32	20	6	58.66	57.5633	47.66	48.67	81.74	81.25
14	32	20	6	56.5	57.5633	49.59	48.67	81.0	81.25
15	32	20	6	57.53	57.5633	48.76	48.67	81.01	81.25

**Table 4:** ANOVA data for electrocoagulation process.

Source		Df	Sum of Squares	Mean Square	F-Ratio	*p*-Value
Cu-Fe Couple						
Chloride	K_1_	1	0.567112	0.567112	0.02	0.8852
	K_2_	1	259.692	259.692	10.56	0.0227
	K_3_	1	44.6985	44.6985	1.82	0.2355
	K_1_K_1_	1	72.8023	72.8023	2.96	0.1460
	K_1_K_2_	1	24.7009	24.7009	1.00	0.3623
	K_1_K_3_	1	0.004225	0.004225	0.00	0.9901
	K_2_K_2_	1	8.11954	8.11954	0.33	0.5905
	K_2_K_3_	1	38.1924	38.1924	1.55	0.2679
	K_3_K_3_	1	64.5006	64.5006	2.62	0.1663
	Total error	5	122.989	24.5978		
	Total (corr.)	14	647.575			
Magnesium	K_1_	1	295.488	295.488	5.08	0.0739
	K_2_	1	544.005	544.005	9.35	0.0282
	K_3_	1	36.4231	36.4231	0.63	0.4646
	K_1_K_1_	1	346.336	346.336	5.95	0.0586
	K_1_K_2_	1	0.570025	0.570025	0.01	0.9250
	K_1_K_3_	1	167.573	167.573	2.88	0.1504
	K_2_K_2_	1	14.2931	14.2931	0.25	0.6411
	K_2_K_3_	1	1.21	1.21	0.02	0.8910
	K_3_K_3_	1	30.7852	30.7852	0.53	0.4995
	Total error	5	290.823	58.1646		
	Total (corr.)	14	1739.64			
Sodium	K_1_	1	15.0426	15.0426	1.03	0.3574
	K_2_	1	42.5503	42.5503	2.90	0.1490
	K_3_	1	37.932	37.932	2.59	0.1685
	K_1_K_1_	1	55.3351	55.3351	3.78	0.1096
	K_1_K_2_	1	134.908	134.908	9.21	0.0289
	K_1_K_3_	1	13.0321	13.0321	0.89	0.3889
	K_2_K_2_	1	21.0247	21.0247	1.44	0.2846
	K_2_K_3_	1	11.2896	11.2896	0.77	0.4202
	K_3_K_3_	1	37.7207	37.7207	2.58	0.1695
	Total error	5	73.2393	14.6479		
	Total (corr.)	14	449.082			

**Table 5:** Optimum operating values for Cu–Fe electrode couple.

	Chloride	Magnesium	Sodium
Stream density (mA/cm^2^)	32	48	32
Contact time (min)	30	10	30
pH	6	6	10
Model prediction results (%)	81	83.28	95
Experimental results (%)	70.65	65.48	86.74

**Table 6:** Comparison of the results in this study with some other research studies on wastewater treatment.

Electrode	Water Types	Responses	Operating Parameters					R_e_ (%)	References
			C_0_ (mg/L)	Conductivity (mS/cm)	j (mA/cm^2^)	pH_i_	t (min)		
**Fe-Al**	**real**	**Cu,** **Ni,** **COD,**	77.5 23.1 32,350	>10.0	32 & 16	10 & 4	30	>90	[12]
Cu-SS & Cu-Zinc	real	TSS Turbidity COD TOC	221 309 291 20	1140	25	5	120	14–5 18–2 65–91 13–64	[40]
Fe- Al	real	Oil-grease, COD, chloride	125, 560, 150	0.98	3	8	30	90 88 50	[41]
Cu	real	COD	7150	-	28	7.7	90	67	[42]
Cu-Cu	real	Manganese	0.94	-	Cont.	7–8	147	>91	[43]
Cu-SS	real	COD TSS Turbidity	7226 3999 226.72	-	5.5 (A)	6.9	60 60 60	89–99 92–100 98–100	[44]
Monopolar Fe	real	TDS, Cl, Br, SO_4_	26,263 8498 6 6562	-	0.8 (A)	8	80	91 93 92 90	[45]
Cu	real	COD Color	3682 350 PCU		17.8	6	120	81 83.5	[46]
Combined Al	real	TDS, Cl, Br, SO_4_	26,263 8498.6 6562	38.062	2	8	80	>90	[47]
Cu-Fe Couple	real	Cl^−^, Mg, Na	185,292 37.7 128,567	>100000	32, 48, 32	6, 6, 10	30, 10, 30	71 65 87	In this study

SS: stainless steel; C_0_: concentration; j: stream density; pH_i_: pH; t: operation time; Cont.: continuous; R_e_: removal efficiency, turbidity, NTU.

**Table 7:** Characterization data of raw drilling wastewater.

Parameters	pH	Conductivity mS/cm	Temp. °C	COD mg/L	TSS mg/L	NO_2_ mg/L	Cr^+6^ mg/L	Iron mg/L	Zinc mg/L
**Mean** **Value**	5.46 ± 0.2	>100,000 ± 5.2	24.8 ± 0.5	7500 ± 25	40.9 ± 1.5	0.03 ± 0.05	0.22 ± 0.01	1.03 ± 0.2	2.66 ± 0.3
**Parameters**	**Oil-Grease mg/L**	**Cl^−^** **mg/L**	**NH_4_-N** **mg/L**	**NO_3_** **mg/L**	**B** **mg/L**	**Na^+^** **mg/L**	**Mg^2+^** **mg/L**	**SO_4_^−2^ Sülfat**	**Ca^2+^** **mg/L**
**Mean** **Value**	9.88 ± 0.2	185,292 ± 4.8	0.181 ± 0.04	0.558 ± 0.2	0.92 ± 0.1	128,567 ± 5.1	37.7 ± 0.5	3058 ± 2.2	1335 ± 1.2

## Data Availability

Data are contained within the article.
